# KYNA analogue SZR72 modifies CFA-induced dural inflammation- regarding expression of pERK1/2 and IL-1β in the rat trigeminal ganglion

**DOI:** 10.1186/s10194-016-0654-5

**Published:** 2016-07-05

**Authors:** M. Lukács, K. Warfvinge, L. S. Kruse, J. Tajti, F. Fülöp, J. Toldi, L. Vécsei, L. Edvinsson

**Affiliations:** Department of Clinical Sciences, Division of Experimental Vascular Research, Lund University, Lund, Sweden; Department of Clinical Experimental Research, Copenhagen University, Glostrup Hospital, Copenhagen, Denmark; Department of Neurology, University of Szeged, Szeged, Hungary; Institute of Pharmaceutical Chemistry and MTA-SZTE Research Group for Stereochemistry, University of Szeged, Szeged, Hungary; Department of Physiology, Anatomy and Neuroscience, University of Szeged, Szeged, Hungary; MTA SZTE Neuroscience Research Group, Szeged, Hungary; Department of Medicine, Institute of Clinical Sciences, Lund University, Sölvegatan 17, SE 221 84 Lund, Sweden

**Keywords:** Complete Freund’s Adjuvant, Dura mater, Trigeminal ganglion, pERK1/2, IL-1β, KYNA

## Abstract

**Background:**

Neurogenic inflammation has for decades been considered an important part of migraine pathophysiology. In the present study, we asked the question if administration of a novel kynurenic acid analogue (SZR72), precursor of an excitotoxin antagonist and anti-inflammatory substance, can modify the neurogenic inflammatory response in the trigeminal ganglion.

**Methods:**

Inflammation in the trigeminal ganglion was induced by local dural application of Complete Freunds Adjuvant (CFA). Levels of phosphorylated MAP kinase pERK1/2 and IL-1β expression in V1 region of the trigeminal ganglion were investigated using immunohistochemistry and Western blot.

**Findings:**

Pretreatment with one dose of SZR72 abolished the CFA-induced pERK1/2 and IL-1β activation in the trigeminal ganglion. No significant change was noted in case of repeated treatment with SZR72 as compared to a single dose.

**Conclusions:**

This is the first study that demonstrates that one dose of KYNA analog before application of CFA can give anti-inflammatory response in a model of trigeminal activation, opening a new line for further investigations regarding possible effects of KYNA derivates.

## Background

Neurogenic inflammation (NI) has for decades been considered an important part of migraine pathophysiology [1]. Basic studies of NI show that it is characterized by proinflammatory responses, caused by the stimulation of peripheral terminals of the primary sensory neurons located in the trigeminal ganglion [2], ultimately involved in sensitization and allodynia. Despite growing interest on the role of neuro-immune interactions in migraine, studies show controversial results regarding serum cytokine levels [3–5].

An interaction between the kynurenine pathway and the immune system has been suggested [6]; the kynureninine system by itself can be activated by inflammatory agents and kynurenic acid has a clear antiinflammatory effect [7]. One of the first studies demonstrating that the kynurenine pathway has a central role in migraine, was performed by Knyihár-Csillik and coworkers, revealed that electrical stimulation of the trigeminal ganglion decreased kynurenine-aminotransferase immunoreactivity in rat dura mater [8]. Recent studies strengthen the importance of the kynurenine system in case of primary headaches, showing significant reduction in levels kynurenic acid in patients with chronic migraine [9–11].

In order to advance our understanding we have developed a method to study inflammation in the trigeminal ganglion induced by local dural application of Complete Freund’s Adjuvant (CFA) [12].

In the present study we administered a novel kynurenic acid analogue (SZR72), a glutamate antagonist, to demonstrate its ability to modify this trigeminal ganglion response and might therefore represent a future approach to migraine treatment.

## Methods

The present study is based on the animal model of inducing inflammatory response in the trigeminal ganglion via activation of the peripheral branches in the dura mater of the trigeminal neurons [12].

### Synthesis of novel KYNA derivative

The KYNA amide was designed in the Department of Pharmaceutical Chemistry and MTA-SZTE Research Group for Stereochemistry, University of Szeged Hungary. The synthesis was performed by coupling of KYNA and 2-dimethylaminoethylamine, afterwards treatment of ethanolic hydrogen chloride, resulting *N*-(2-*N*,*N*-dimethylaminoethyl)-4-oxo-1H-quinoline-2-carboxamide hydrochloride. The structural properties of SZR72 are the following: presence of a water-soluble side-chain, the inclusion of a new cationic center, and side-chain substitution in order to facilitate brain penetration [6, 13].

### Animals

Adult male Sprague-Dawley rats (220–300 g) (*n* = 49, 24 for immunohistochemistry, 25 for Western blot) were used. The animals were maintained under standard laboratory conditions with free access to food and tap water. The study followed the guidelines of the European Communities Council (86/609/ECC) and approved by the Ethics Committee of The Faculty of Medicine, University of Szeged, Hungary.

### Operations

We have recently described the method in detail [12].

### Treatments

The animals were divided into 5 groups: (i) pre-treatment KYNA (KYNA analog 1 h before CFA administration), (ii) pre-treatment saline (saline 1 h before CFA), (iii) repeated treatment (KYNA analog every 12 h, for 7 days), (iv) repeated saline (saline every 12 h, for 7 days) and (v) fresh (intact, control rats) (Table 1). The KYNA analog (300 mg/kg body weight was dissolved in 1 ml saline) or saline (1 ml) were given intraperitoneally.

**Table 1:** Animal groups used for immunohistochemistry and Western blot

Groups	Treatment 1 h before operation	Treatment every 12 hrs, for 7 days	No of animals, IHC	No of animals, WB
Pre-treatment SZR72	KYNA derivate	-	6	5
Pre-treatment saline	saline	-	4	5
Repeated treatment SZR72	KYNA derivate	KYNA derivate	6	5
Repeated saline	saline	saline	4	5
Intact control	-	-	4	5

As shown before [12] the “inflammatory” response to dural CFA was studied after 1 week, left trigeminal ganglion was removed and the specimens were prepared for immunohistochemistry or Western blot.

### Immunohistochemistry and microscopic analysis

Immunohistochemistry was performed to demonstrate the localization of pERK1/2 and IL-1β, and semi-quanitatively evaluate the alterations in their expression in the trigeminal ganglion. Details of the antibodies are given in Table 2. The immunohistochemistry method and the microscopic analysis were described in our previous study [12].

**Table 2:** Details of primary and secondary antibodies used for IHC and WB

	Name	Product code	Host	Dilution	Company
IHC					
	Phospho-p44/42 MAPK (Erk1/2) (Thr202/Tyr204)	4376	Rabbit	1:50	Cell Signaling Technology, Danvers, MA, USA
	Anti IL-1 beta antibody	ab 9787	Rabbit	1:100	Abcam; Cambridge, UK
WB					
	Phospho-p44/42 MAPK (Erk1/2) (Thr202/Tyr204)	4376	Rabbit	1:1000	Cell Signaling Technology, Danvers, MA, USA
	Anti IL-1 beta antibody	ab 9787	Rabbit	1:500	Abcam; Cambridge, UK

### Western blot

The method used for Western blot is described in one of our studies [14]. Data were normalised to an internal loading control sample to adjust for gel-to-gel variation and both pERK and pro-IL1beta calculated relative to t-ERK.

## Findings

### Immunohistochemistry

In evaluating the immunohistochemical results, the medullary zone of the trigeminal ganglion and the V1 region were chosen.

## pERK1/2

**Fig. 1:**
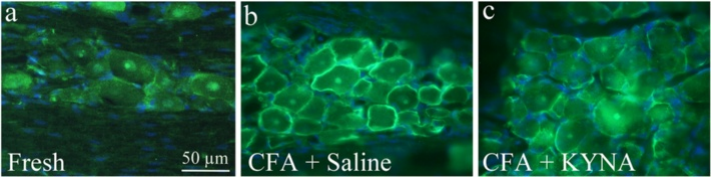
pERK1/2 in the trigeminal ganglion. **a** In fresh animals, pERK1/2 immunoreactivity was detected in a few nuclei of the trigeminal ganglia, including nucleoli, in fresh animals. No immunoreactivity was found in the neuronal cytoplasm. A few SGC were considered as positively stained. **b** In CFA animals repeated treatment with saline i.p., high-intensity pERK1/2 immunoreactivity was observed in SGCs. **c** Animals treated with KYNA for 7 days, diminished immunoreactivity to pERK1/2 in SGC

As described earlier [12], pERK1/2 immunoreactivity was detected in a few nuclei of the neurons, including nucleoli, in fresh animals. A few satellite glial cells (SGC) were considered as positively stained (Fig. 1a). In CFA treated animals i.p. saline revealed high-intensity pERK1/2 immunoreactivity in SGCs, especially in the anteromedial zone of the ganglion (Fig. 1b). No significant difference was noted in case of repeated use of saline i.p.

In contrast, animals treated with the novel KYNA derivate, showed abolished pERK1/2 immunoreactivity in SGC (Fig. 1c). Repeated treatment with the KYNA analog resulted in mitigation of the SGC activation compared to saline treatment; both positive and negative SGC were detected.

Blinded analysis of the SGCs fluorescence intensity showed significant difference between the control groups and the pre-treatment group with KYNA derivate (*p* < 0.05). In contrast, no significant difference between the control groups and repeated treatment with KYNA analog was found (*p* = 0.069), neither between the pre-treatment and repeated treatment groups with KYNA analog (*p* = 0.567).

## IL-1β

**Fig. 2:**
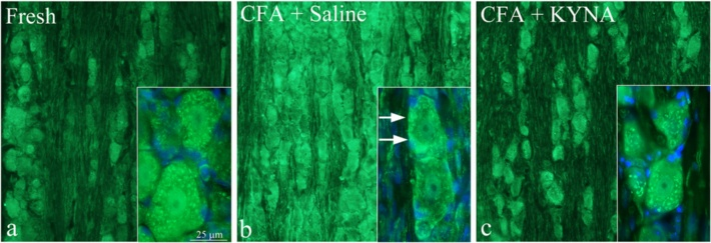
IL-1β in the trigeminal ganglion. **a** IL-1β immunoreactivity was observed in the neuronal cytoplasm (in a granular manner), in a few nuclei and in the nerve fibers of fresh animals. No immunoreactivity was detected in the SGC. **b** After i.p. treatment with saline for 7 days, increased IL-1β immunoreactivity was observed both intracellularly in the neurons, and in the fibers. In addition, a “ring” IL-1β immunoreactivity close to the neuronal cell membrane was evident. **c** Following i.p. treatment with KYNA for 7 days, the homogenous immunoreactivity close to the cell membrane disappeared, returning to the granular cytoplasmatic pattern observed in fresh animals. No difference could be noted in the neuronal nuclei and in fibers, and no immunoreactivity was detected in the SGC

**Fig. 3:**
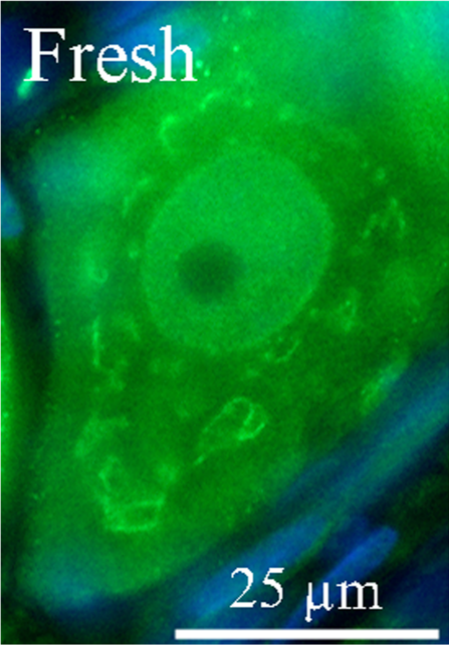
A detailed distribution of IL-1β in a trigeminal ganglion neuron

As described before [12], IL-1β immunoreactivity was observed in the neuronal cytoplasm (in a granular manner), in a few nuclei and in the nerve fibers of fresh animals. No immunoreactivity was detected in the SGC (Figs. 2a and 3). After i.p. treatment with saline and application of CFA, increased IL-1β immunoreactivity was observed both intracellularly in the neurons, and in the fibers. In addition, a “ring” IL-1β immunoreactivity close to the neuronal cell membrane was evident, which differed from the granular pattern seen in fresh animals (Fig. 2b).

Following i.p. treatment with KYNA analogue, the homogenous immunoreactivity close to the cell membrane disappeared, returning to the granular cytoplasmatic pattern observed in fresh animals. Repeated treatment with KYNA derivate showed no difference compared to the pretreatment with KYNA. No difference could be noted in the neuronal nuclei and in fibers, and no immunoreactivity was detected in the SGC (Fig. 2c).

Blinded analysis of the neuronal fluorescence in general, but also the presence of homogenous immunofluorescence close to the cell membrane, showed significant difference between control groups (pre-treatment and repeated treatment with saline) and pre-treatment with KYNA analog (*p* < 0.05). IL-1β immunoreactivity was significantly decreased in the repeated treatment KYNA group compared to control groups treated with saline (*p* < 0.001). Between the pre-treated and repeated treatment groups, no significant difference could be noted (*p* = 0.969).

**Fig. 4:**
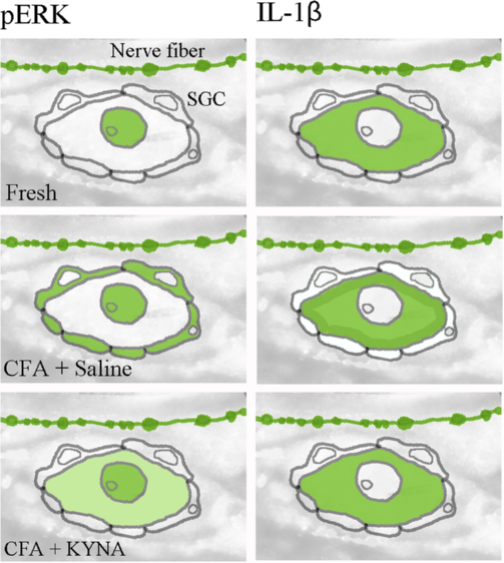
A schematic drawing of the distribution of pERK1/2 and IL-1β

A schematic drawing of the main results for the immunohistochemistry is shown in Fig. 4 and results are summarized in Table 3.

**Table 3:** Summary of results for IHC for pERK and IL-1β regarding neurons, fibers and satellite glial cells (SGC); intensity scale: “-” no staining, “+/-” very weak staining, “+”-weak staining, “++”-moderate staining, “+++” strong staining

	pERK 1/2			IL-1β		
Groups	Neurons (nucleolei)	Fibers	SGC	Neurons	Fibers	SGC
Pre-treatment SZR72 (dura exposed to CFA)	+	+	+/-	+/-	+	-
Pre-treatment saline (dura exposed to CFA)	+	+	+++	+++	+	-
Repeated treatment SZR72 (dura exposed to CFA)	+	+	++	+/-	+	-
Repeated treatment saline (dura exposed to CFA)	+	+	+++	+++	+	-
Intact control (unoperated)	+	+	+/-	-	+	-

### Western blot

**Fig. 5:**
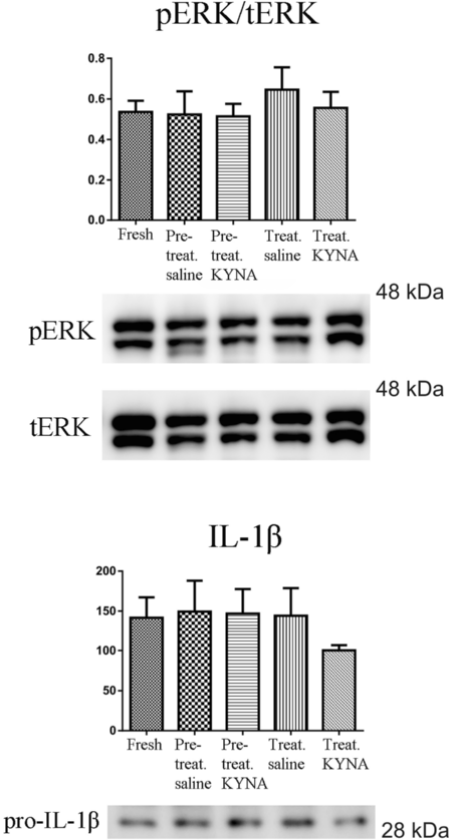
Western blot of pERK, t-ERK and IL-1β in the whole trigeminal ganglion revealing no significant difference between the treatment groups

Staining for both pERK/tERK and pro-IL-1β using tissue lysates of complete left trigeminal ganglia did not reveal any significant changes in expression levels between treatment groups as analyzed by one-way ANOVA (*p* = 0.8282 and *p* = 0.7461, respectively) with Bonferroni multiple correction post-testing and Student’s t-test (Fig. 5).

## Discussion

The main question asked was if the KYNA derivate could modify the CFA-induced long-term inflammatory activation of the trigeminal ganglion, shown by increased expression of pERK1/2 and IL-1β [12].

The role of the kynurenine pathway in the CNS is very complex, modulating several neurotransmitters. In 1947 Beadle at al. [15] discovered that the major route for tryptophan metabolism to nictoniamide and its conjugates is the kynurenine pathway. Tryptophan represents a precursor of serotonin, a neurotransmitter playing an important role in the migraine pathophysiology [16]. The kynurenine pathway, having several neuroactive metabolites including kynurenic acid (KYNA) [17, 18], has an important role in various diseases of the CNS [6, 19]. Astrocytes represent one source of neuroprotective KYNA [6, 20]; KYNA was presumed to have protective effect in neuronal cell death [21, 22]. Studies have also suggested that an elevated extracellular KYNA level would be needed to act more effectively [23], leading to the idea of systemic administration of KYNA. This was not proved to be an ideal therapeutic option, as KYNA poorly penetrates the blood-brain barrier and it undergoes a rapid clearance from the brain and the circulation [24]. To overcome these difficulties new KYNA analogues were synthesized to facilitate blood-brain penetration [13]. It has been shown that the analogue SZR72 has similar neuroprotective effect as KYNA and cross more easily the BBB [25, 26].

In the present study SZR72 (1 mmol/kg bodyweight) reduced the CFA- elevated response on pERK1/2 and IL-1β activation in the trigeminal ganglion. The experiments were designed to answer the question if in case of chronification of migraineous mechanism (i) one dose of SZR72 is enough to attenuate the activation or if (ii) daily treatment would be needed.

The pERK1/2 is suggested to represent a rapid and robust marker of activation and inflammation [27, 28], whereas IL-1β is a late marker of maintained neuropathic pain [29]. In case of using one dose of SZR72, pERK1/2 activation in the SGC was significantly diminished, whereas in case of repeated treatment the difference was not found to be significant. This finding might be explained by the property of pERK1/2 as being an early marker and not an optimal sign of prolonged activation.

The IL-1β activation was modified both after one dose of SZR72 and after repeated treatment with SZR72. Although no significant difference was observed between the pre-treatment and repeated treatments in case of IL-1β, there was a significant difference between the repeated SZR72 treated group and control groups. IL-1β might represent a more proper marker to examine long term effects of SZR72.

When it comes to our Western blot studies, the same tendency can be followed, without any significant difference between the groups. This might be explained by a methodological problem: to isolate macroscopically the V1 region is almost impossible therefor**e** the whole ganglia were processed in case of WB studies. We might reasonably postulate that our WB studies are not specific enough for the V1 region of the trigeminal ganglion.

In conclusion, this is the first study to address the question whether daily use of the new KYNA analog would be more effective than one dose prior activation in a chronification model of trigeminal activation. Pretreatment with one dose was able to abolish pERK and IL-1β activation in the trigeminal ganglion. These findings open a new line for further investigations which could result in a new way to modulate inflammation in chronic migraine.

## References

[1] Markowitz S, Saito K, Moskowitz MA (1987). Neurogenically mediated leakage of plasma protein occurs from blood vessels in dura mater but not brain. J Neurosci.

[2] Geppetti P, Nassini R, Materazzi S, Benemei S (2008). The concept of neurogenic inflammation. BJU Int.

[3] Goadsby PJ, Edvinsson L, Ekman R (1990). Vasoactive peptide release in the extracerebral circulation of humans during migraine headache. Ann Neurol.

[4] Goadsby PJ, Edvinsson L, Ekman R (1988). Release of vasoactive peptides in the extracerebral circulation of humans and the cat during activation of the trigeminovascular system. Ann Neurol.

[5] Perini F, D’Andrea G, Galloni E, Pignatelli F, Billo G, Alba S (2005). Plasma cytokine levels in migraineurs and controls. Headache.

[6] Vecsei L, Szalardy L, Fulop F, Toldi J (2013). Kynurenines in the CNS: recent advances and new questions. Nat Rev Drug Discov.

[7] Mandi Y, Vecsei L (2012). The kynurenine system and immunoregulation. J Neural Transm (Vienna).

[8] Knyihar-Csillik E, Chadaide Z, Okuno E, Krisztin-Peva B, Toldi J, Varga C (2004). Kynurenine aminotransferase in the supratentorial dura mater of the rat: effect of stimulation of the trigeminal ganglion. Exp Neurol.

[9] Curto M, Lionetto L, Negro A, Capi M, Perugino F, Fazio F (2015). Altered serum levels of kynurenine metabolites in patients affected by cluster headache. J Headache Pain.

[10] Curto M, Lionetto L, Negro A, Capi M, Fazio F, Giamberardino MA (2015). Altered kynurenine pathway metabolites in serum of chronic migraine patients. J Headache Pain.

[11] Curto M, Lionetto L, Fazio F, Mitsikostas DD, Martelletti P (2015). Fathoming the kynurenine pathway in migraine: why understanding the enzymatic cascades is still critically important. Intern Emerg Med.

[12] Lukacs M, Haanes KA, Majlath Z, Tajti J, Vecsei L, Warfvinge K (2015). Dural administration of inflammatory soup or Complete Freund’s Adjuvant induces activation and inflammatory response in the rat trigeminal ganglion. J Headache Pain.

[13] Fulop F, Szatmari I, Toldi J, Vecsei L (2012). Modifications on the carboxylic function of kynurenic acid. J Neural Transm (Vienna).

[14] Skovsted GF, Kruse LS, Larsen R, Pedersen AF, Trautner S, Sheykhzade M (2014). Heart ischaemia-reperfusion induces local up-regulation of vasoconstrictor endothelin ETB receptors in rat coronary arteries downstream of occlusion. Br J Pharmacol.

[15] Beadle GW, Mitchell HK, Nyc JF (1947). Kynurenine as an Intermediate in the Formation of Nicotinic Acid from Tryptophane by Neurospora. Proc Natl Acad Sci U S A.

[16] Pardutz A, Fejes A, Bohar Z, Tar L, Toldi J, Vecsei L (2012). Kynurenines and headache. J Neural Transm (Vienna).

[17] Amori L, Guidetti P, Pellicciari R, Kajii Y, Schwarcz R (2009). On the relationship between the two branches of the kynurenine pathway in the rat brain in vivo. J Neurochem.

[18] Vamos E, Pardutz A, Klivenyi P, Toldi J, Vecsei L (2009). The role of kynurenines in disorders of the central nervous system: possibilities for neuroprotection. J Neurol Sci.

[19] Guillemin GJ, Cullen KM, Lim CK, Smythe GA, Garner B, Kapoor V (2007). Characterization of the kynurenine pathway in human neurons. J Neurosci.

[20] Guillemin GJ, Kerr SJ, Smythe GA, Smith DG, Kapoor V, Armati PJ (2001). Kynurenine pathway metabolism in human astrocytes: a paradox for neuronal protection. J Neurochem.

[21] Lee do Y, Lee KS, Lee HJ, Noh YH, Kim do H, Lee JY (2008). Kynurenic acid attenuates MPP(+)-induced dopaminergic neuronal cell death via a Bax-mediated mitochondrial pathway. Eur J Cell Biol.

[22] Carrillo-Mora P, Mendez-Cuesta LA, Perez-De La Cruz V, Fortoul-van Der Goes TI, Santamaria A (2010). Protective effect of systemic L-kynurenine and probenecid administration on behavioural and morphological alterations induced by toxic soluble amyloid beta (25-35) in rat hippocampus. Behav Brain Res.

[23] Urenjak J, Obrenovitch TP (2000). Kynurenine 3-hydroxylase inhibition in rats: effects on extracellular kynurenic acid concentration and N-methyl-D-aspartate-induced depolarisation in the striatum. J Neurochem.

[24] Fukui S, Schwarcz R, Rapoport SI, Takada Y, Smith QR (1991). Blood-brain barrier transport of kynurenines: implications for brain synthesis and metabolism. J Neurochem.

[25] Marosi M, Nagy D, Farkas T, Kis Z, Rozsa E, Robotka H (2010). A novel kynurenic acid analogue: a comparison with kynurenic acid. An in vitro electrophysiological study. J Neural Transm (Vienna).

[26] Zadori D, Nyiri G, Szonyi A, Szatmari I, Fulop F, Toldi J (2011). Neuroprotective effects of a novel kynurenic acid analogue in a transgenic mouse model of Huntington’s disease. J Neural Transm (Vienna).

[27] Iwashita T, Shimizu T, Shibata M, Toriumi H, Ebine T, Funakubo M (2013). Activation of extracellular signal-regulated kinase in the trigeminal ganglion following both treatment of the dura mater with capsaicin and cortical spreading depression. Neurosci Res.

[28] Gao YJ, Ji RR (2009). c-Fos and pERK, which is a better marker for neuronal activation and central sensitization after noxious stimulation and tissue injury?. Open Pain J.

[29] Ruohonen S, Khademi M, Jagodic M, Taskinen HS, Olsson T, Roytta M (2005). Cytokine responses during chronic denervation. J Neuroinflammation.

